# Editorial: Cancer in people living with HIV/AIDS

**DOI:** 10.3389/fcimb.2025.1575386

**Published:** 2025-03-13

**Authors:** DeGaulle I. Chigbu, Pooja Jain

**Affiliations:** ^1^ Pennsylvania College of Optometry, Salus at Drexel University, Elkins Park, PA, United States; ^2^ Department of Microbiology and Immunology, Drexel University College of Medicine, Philadelphia, PA, United States

**Keywords:** cancer, HIV, immunosuppression, immunotherapy, oncogenic virus

Human immunodeficiency virus (HIV), a member of the Retroviridae family, is an enveloped virus with a positive-sense single-stranded RNA genome (Hoffman et al., 2020). The RNA genome is reverse transcribed into a proviral double-stranded DNA (dsDNA) by reverse transcriptase, and the proviral dsDNA is subsequently integrated into the host genome by integrase (Tekeste et al., 2015). HIV usually infects CD4-expressing immune cells, such as CD4^+^T cells, macrophages, and dendritic cells, leading to immunodeficiency (Hoffman et al., 2020). People living with HIV (PLWH) are susceptible to developing additional complications (i.e., cancer and opportunistic infections) since HIV infects and destroys the activators and controllers of the immune system (Manches et al., 2014; Vidya Vijayan et al., 2017). It is important to note that a dysfunctional immune surveillance system in PLWH makes it possible for cancer cells to evade immune recognition and destruction (Puronen et al., 2019), resulting in non-Hodgkin’s lymphomas (NHLs) such as diffuse large B-cell lymphoma (DLBCL) and Burkitt lymphoma that represent the major AIDS defining cancers.

PLWH are at significant risk of developing cancer due to the reactivation of dormant cancer-causing viruses despite the patient’s compliance with antiretroviral therapy (Javid et al., 2023). Many cancers in PLWH are due to oncogenic viruses such as Hepatitis B virus (HBV), HCV, Human herpesvirus-8 (HHV-8), Human papillomavirus (HPV), Epstein-Barr virus (EBV), Kaposi sarcoma-associated herpesvirus (KSHV), and Merkel cell polyomavirus (MCPyV) (Lurain, 2023). Human oncogenic viruses can promote uncontrolled cellular proliferation and inhibit apoptosis of infected cells, resulting in the development of cancer (Krump and You, 2018). The chronic immune activation and chronic inflammation associated with persistent infection from HIV and oncogenic viruses play a role in the development of cancer (Senba and Mori, 2012; Paiardini and Muller-Trutwin, 2013; Odeny et al., 2024; Shiels et al., 2018; Zamaraev et al., 2020). Kaposi sarcoma, breast cancer, lung cancer, liver cancer, prostate cancer, non-Hodgkin lymphoma, conjunctival cancer, cervical cancer, laryngeal cancer, cutaneous squamous cell carcinoma, colorectal cancer, oropharyngeal cancer, melanoma, and laryngeal cancer have been observed in HIV seropositive individuals (Sengayi-Muchengeti et al., 2023; Lurain, 2023; Odeny et al., 2024). The high burden of cancer in PLWH co-infected with oncogenic viruses is a compelling reason for continued research in targeted therapy for these HIV-associated cancers (Lurain, 2023; Odeny et al., 2024).

This editorial will present articles published on the Research Topic, highlighting the link between cancer and PLWH. Vaughan et al. discussed the immunological abnormalities and prognostic relevance of immune mediators in PLWH with DLBCL. These researchers studied 76 individuals with DLBCL, of which 61 were PLWH. In this study, the researchers found that these patients have significant levels of immune mediators such as interleukin-6 and C-reactive protein. The authors suggested that the derangement of immune mediators is common in PLWH with DLBCL. Morsica et al. reported two cases of PLWH with unresectable hepatocellular carcinoma who developed intolerance to Lenvatinib. The authors suggested that PLWH with unresectable hepatocellular carcinoma have reduced overall survival outcomes, and a multidisciplinary approach is needed to manage these patients. Lenvatinib, a multi-targeted receptor tyrosine kinase inhibitor, is an FDA-approved anti-cancer therapy indicated for patients with unresectable hepatocellular carcinoma (Hao and Wang, 2020). Kaposi sarcoma, caused by KSHV, is a multifocal proliferative vascular tumor observed in PLWH. Julius et al. discussed a case of an individual with HIV seropositivity co-infected with KSHV and EBV in a conjunctival Kaposi sarcoma. The authors recommended additional studies to determine whether this presentation of KSHV and EBV co-localization of a Kaposi sarcoma tumor can contribute to the development and progression of Kaposi sarcoma cancer. Xiong et al. analyzed the safety and efficacy of PD-1 inhibitors, an immune checkpoint inhibitor, for treating cancer in PLWH. In a study of 16 PLWH with cancer, the authors demonstrated that monoclonal antibodies against PD1 and PD-L1 are safe and efficacious cancer immunotherapeutic agents. Thus, camrelizumab and other anti-PD-1/anti-PD-L1 are safe and efficacious in PLWH with advanced cancer (Baraibar et al., 2019). Because PLWH are at risk of being an underserved group with limited access to cancer clinical trials, it is crucial to include these patients in cancer clinical trials (Uldrick et al., 2017). Yang et al. evaluated eight studies involving 2180 patients, and they revealed that PLWH with colorectal cancer had a poor overall postoperative survival outcome. These authors identified that higher HIV viral load and lower CD4^+^T cell count is contributing factors to the poor postoperative overall survival in PLWH. Because PLWH with colorectal cancer have a worse prognosis, there is a need to provide these patients a multidisciplinary approach to cancer therapy involving infectious disease specialists and immuno-oncologists. In their commentary, Sun et al. discussed the efficacy and adverse reactions associated with Lenvatinib. The authors discussed the tolerability and safety of Lenvatinib in treating patients with hepatocellular carcinoma (HCC). It has been revealed that patients with advanced HCC had a better overall survival outcome when treated with Lenvatinib (Jaiswal et al., 2023). Hua et al (Hua et al., 2024). showed that patients with HCC who used Lenvatinib had better overall survival than those on Sorafenib. The authors recommended that oncology surveillance and a multidisciplinary approach to managing PLWH with HCC will be beneficial in reducing the cancer burden in PLWH.

In conclusion, the papers published in this Research Topic have provided insight into cancer in people living with HIV as well as reiterated the necessity of instituting a multidisciplinary approach to managing PLWH with cancer. Additionally, inclusion of these individuals with HIV seropositivity in clinical trials on cancer immunotherapy is very crucial to ensure that cancer immunotherapies are safe and efficacious in PLWH with cancer. We express our appreciation to all authors who contributed to this Research Topic, and we hope that this Research Topic will continue to expand our knowledge of cancer in people living with HIV.

## References

[B1] Baraibar I. Melero I. Ponz-Sarvise M. Castanon E. (2019). Safety and tolerability of immune checkpoint inhibitors (PD-1 and PD-L1) in cancer. Drug Saf. 42, 281–294. doi: 10.1007/s40264-018-0774-8 30649742

[B2] Hao Z. Wang P. (2020). Lenvatinib in management of solid tumors. Oncologist 25, e302–e310. doi: 10.1634/theoncologist.2019-0407 32043789 PMC7011622

[B3] Hoffman M. Chigbu D. I. Crumley B. L. Sharma R. Pustylnikov S. Crilley T.. (2020). “Human acute and chronic viruses: host-pathogen interactions and therapeutics,” in Advanced concepts in human immunology: prospects for disease control, 1–120. Cham Switzerland: Springer Nature.

[B4] Hua X. Yin Z. Liang J. Chen W. Gong H. (2024). Efficacy and safety comparison between Lenvatinib and Sorafenib in hepatocellular carcinoma treatment: a systematic review and meta-analysis of real-world study. Eur. J. Gastroenterol. Hepatol. 36, 120–128. doi: 10.1097/MEG.0000000000002668 37942731 PMC10695342

[B5] Jaiswal V. Hameed M. Naz S. Roy P. Deb N. Ukrani J.. (2023). Efficacy of lenvatinib versus sorafenib in the primary treatment of advanced hepatocellular carcinoma: A meta-analysis. JGH Open 7, 832–840. doi: 10.1002/jgh3.12999 38162860 PMC10757498

[B6] Javid H. Sharbaf Mashhad A. Yazdani S. Akbari Oryani M. Akbari S. Rezagholinejad N.. (2023). The role of viruses in cancer development versus cancer therapy: An oncological perspective. Cancer Med. 12, 11127–11148. doi: 10.1002/cam4.v12.10 36880311 PMC10242333

[B7] Krump N. A. You J. (2018). Molecular mechanisms of viral oncogenesis in humans. Nat. Rev. Microbiol. 16, 684–698. doi: 10.1038/s41579-018-0064-6 30143749 PMC6336458

[B8] Lurain K. (2023). Treating cancer in people with HIV. J. Clin. Oncol. 41, 3682–3688. doi: 10.1200/JCO.23.00737 37267514 PMC10351946

[B9] Manches O. Frleta D. Bhardwaj N. (2014). Dendritic cells in progression and pathology of HIV infection. Trends Immunol. 35, 114–122. doi: 10.1016/j.it.2013.10.003 24246474 PMC3943663

[B10] Odeny T. A. Fink V. Muchengeti M. Gopal S. (2024). Cancer in people with HIV. Infect. Dis. Clin. North Am. 38, 531–557. doi: 10.1016/j.idc.2024.06.007 39111924 PMC11529824

[B11] Paiardini M. Muller-Trutwin M. (2013). HIV-associated chronic immune activation. Immunol. Rev. 254, 78–101. doi: 10.1111/imr.2013.254.issue-1 23772616 PMC3729961

[B12] Puronen C. E. Ford E. S. Uldrick T. S. (2019). Immunotherapy in people with HIV and cancer. Front. Immunol. 10, 2060. doi: 10.3389/fimmu.2019.02060 31555284 PMC6722204

[B13] Senba M. Mori N. (2012). Mechanisms of virus immune evasion lead to development from chronic inflammation to cancer formation associated with human papillomavirus infection. Oncol. Rev. 6, e17. doi: 10.4081/oncol.2012.e17 25992215 PMC4419623

[B14] Sengayi-Muchengeti M. Singh E. Chen W. C. Bradshaw D. De Villiers C. B. Newton R.. (2023). Thirteen cancers associated with HIV infection in a Black South African cancer patient population, (1995-2016). Int. J. Cancer 152, 183–194. doi: 10.1002/ijc.34236 36054877

[B15] Shiels M. S. Islam J. Y. Rosenberg P. S. Hall H. I. Jacobson E. Engels E. A. (2018). Projected cancer incidence rates and burden of incident cancer cases in HIV-infected adults in the United States through 2030. Ann. Intern. Med. 168, 866–873. doi: 10.7326/M17-2499 29801099 PMC6329294

[B16] Tekeste S. S. Wilkinson T. A. Weiner E. M. Xu X. Miller J. T. Le Grice S. F.. (2015). Interaction between reverse transcriptase and integrase is required for reverse transcription during HIV-1 replication. J. Virol. 89, 12058–12069. doi: 10.1128/JVI.01471-15 26401032 PMC4645309

[B17] Uldrick T. S. Ison G. Rudek M. A. Noy A. Schwartz K. Bruinooge S.. (2017). Modernizing clinical trial eligibility criteria: recommendations of the american society of clinical oncology-friends of cancer research HIV working group. J. Clin. Oncol. 35, 3774–3780. doi: 10.1200/JCO.2017.73.7338 28968173 PMC5793223

[B18] Vidya Vijayan K. K. Karthigeyan K. P. Tripathi S. P. Hanna L. E. (2017). Pathophysiology of CD4+ T-cell depletion in HIV-1 and HIV-2 infections. Front. Immunol. 8, 580. doi: 10.3389/fimmu.2017.00580 28588579 PMC5440548

[B19] Zamaraev A. V. Zhivotovsky B. Kopeina G. S. (2020). Viral infections: negative regulators of apoptosis and oncogenic factors. Biochem. (Mosc) 85, 1191–1201. doi: 10.1134/S0006297920100077 PMC7590567 33202204

